# ZnO Nanorods with High Photocatalytic and Antibacterial Activity under Solar Light Irradiation

**DOI:** 10.3390/ma11112158

**Published:** 2018-11-01

**Authors:** Faouzi Achouri, Christophe Merlin, Serge Corbel, Halima Alem, Laurence Mathieu, Lavinia Balan, Ghouti Medjahdi, Myriam Ben Said, Ahmed Ghrabi, Raphaël Schneider

**Affiliations:** 1 Université de Lorraine, CNRS, LRGP, F-54000 Nancy, France; achouri87@gmail.com (F.A.); serge.corbel@univ-lorraine.fr (S.C.); 2 Centre de Recherches et Technologies des Eaux (CERTE), Laboratoire Eaux Usées et Environnement, P.O. Box 273, Soliman, Tunis 8020, Tunisia; myriam_rebia@yahoo.fr (M.B.S.); a.ghrabi@yahoo.fr (A.G.); 3 Faculté des Sciences de Bizerte, Université de Carthage, Jarzouna, Bizerte 7021, Tunisia; 4 Université de Lorraine, CNRS, LCPME, F-5v4000 Nancy, France; christophe.merlin@univ-lorraine.fr (C.M.); laurence.mathieu@univ-lorraine.fr (L.M.); 5 Université de Lorraine, CNRS, IJL, F-54000 Nancy, France; halima.alem@univ-lorraine.fr (H.A.); ghouti.medjahdi@univ-lorraine.fr (G.M.); 6 EPHE, PSL Research University, LCPME, UMR 7564 Nancy, France; 7 IS2M, CNRS UMR 7361, 15 Rue Jean Starcky, 68093 Mulhouse, France; lavinia.balan@uha.fr

**Keywords:** ZnO, photocatalysis, immobilized catalyst, *Escherichia coli*, bacterial decontamination

## Abstract

ZnO nanorods (NRs) with an average length and diameter of 186 and 20 nm, respectively, were prepared through a mild solvothermal route and used as photocatalysts either as dispersed powder or immobilized on glass slides. The ZnO NRs were characterized by scanning electron microscopy (SEM), transmission electron microscopy (TEM), and X-ray diffraction (XRD). Dispersed ZnO NRs and, to a lesser extent, immobilized ZnO NRs were demonstrated to exhibit high photocatalytic activity under simulated sunlight of low intensity (5.5 mW/cm^2^) both for the degradation of the Orange II dye and for *Escherichia coli* bacterial decontamination (2.5-fold survival decrease after 180 min irradiation for immobilized NRs). SEM, atomic force microscopy (AFM), fluorescence spectroscopy, and epifluorescence microscopy demonstrate that cell surface damages are responsible of bacterial inactivation. The immobilized ZnO NRs could be reused up to five times for bacterial decontamination at comparable efficiency and therefore have great potential for real environmental applications.

## 1. Introduction

Since the first report of bacterial photocatalytic inactivation in 1985 [1], photocatalysis has gained considerable interest regarding the decontamination of numerous pathogenic microorganisms, such as bacteria, viruses, fungi, and protozoa, in drinking and wastewater [2,3,4,5,6,7]. Once the photocatalyst is activated by light with an energy equal to or greater than the bandgap of the semiconductor, electron (e^−^)/hole (h^+^) pairs are generated in the conduction and valence bands, respectively. Next, some of these pairs migrate to the photocatalyst surface. In the primary stage, e^−^ react with O_2_ adsorbed at the photocatalyst surface to generate superoxide O_2_^•−^ radicals, while h^+^ react with water to produce hydroxyl ^•^OH radicals. These reactive oxygen species (ROS) are powerful oxidants able to mineralize organic pollutants and also affect the integrity of microorganisms, from their cell envelope to intracellular components such as proteins and DNA [2,3,4,5,6,7]. 

Zinc oxide (ZnO) is an n-type direct bandgap semiconductor (*Eg* = 3.3 eV) and has a large exciton binding energy (60 meV at room temperature). Due to its attractive optical and electronic properties, ZnO has found applications in solar cells, optical coatings, and electrical devices [8,9,10,11]. Although ZnO exhibits a slightly lower photostability than TiO_2_ [12], it has also been demonstrated as being one of the most promising materials for photocatalytic applications such as degradation of organic pollutants and microorganism decontamination [13,14,15]. The native defects present in ZnO, such as oxygen vacancies and zinc interstitials, not only allow the decrease of e^−^/h^+^ pair recombinations but also the increase of visible light absorption [16,17]. Moreover, due to its good physical and chemical stability, ZnO possesses a photocatalytic activity comparable to that of TiO_2_. Another interesting aspect of ZnO, especially for photocatalytic applications, is that various nanostructures with different morphologies (spheres, rods, tubes, needles, etc.) can be engineered using relatively simple and mild methods (sol–gel, solvothermal, etc.) without any templates or surfactants. Numerous reports have demonstrated that the photocatalytic efficiency depends on ZnO particle size and morphology [18,19]. One-dimensional nanostructures, like nanorods, formed by electrostatic interactions between ZnO clusters [20,21,22], generally exhibit a higher photocatalytic activity than spherical ZnO particles due to their high surface area that allows more light to be trapped, thus generating more charge carriers [18,19,22,23,24,25,26]. Moreover, due to their dimensional anisotropy, more e^−^ and h^+^ exist on active sites at the catalyst surface, leading to increased ROS generation.

Recently, we described the synthesis and the photocatalytic activity of small-sized undoped and doped ZnO rods that have a good potential for water decontamination due to the presence of defect sites that favor light absorption and hinder e^−^/h^+^ recombination and to their high specific surface area [22,23,27]. However, the facile recovery, handling, and reuse of the photocatalyst after water decontamination is the stumbling block for the development and wide use of this photocatalysis technology. In that context, the use of immobilized photocatalysts can be one alternative. Moreover, immobilization avoids working with small particles that are subject to agglomeration and also to the release of ZnO in the environment. The main drawback associated with particle immobilization is the decrease of the catalyst specific surface area and thus of its reactivity [28,29]. This hurdle might be overcome through the use of nanosized ZnO particles with a high surface-to-volume ratio in which a high density of active sites for adsorption and photodegradation can be maintained.

In this paper, we report the preparation of ZnO nanorods (NRs) with an average length and diameter of ca. 186 and 20 nm, respectively, and their successful immobilization on glass slides by a mild thermal treatment at 70 °C. The photocatalytic activity of ZnO NRs, both in dispersed and immobilized forms, was explored under simulated sunlight irradiation through the degradation of the Orange II dye used as model pollutant. ZnO NRs also exhibited excellent photoinactivation activity toward *Escherichia coli* cells, and the immobilized ZnO photocatalyst could be reused up to five times for bacterial inactivation without significant loss of its activity, indicating its great potential for water treatment. 

## 2. Materials and Methods 

ZnO NRs were prepared via a solvothermal method according to reference [30]. The full synthetic protocol and the methods used to characterize these particles are provided in the Supplementary Materials.

### 2.1. Photocatalytic Degradation of Orange II

The photocatalytic activity of ZnO NRs was evaluated by the degradation of an aqueous solution of the Orange II dye (10 mg/L) at 25 °C under simulated sunlight irradiation produced by Sylvania LuxLine FHO T5 neon tubes (see Figure S1 for the UV–visible emission spectrum of the tubes used). The intensity at the surface of the dye solution was estimated to be 5.5 mW/cm^2^. In the case of dispersed ZnO NRs, the photocatalyst (40 mg) was dispersed in 40 mL of Orange II solution and the suspension was stirred for 30 min in the dark to achieve an adsorption–desorption equilibrium. Under stirring, the suspension was exposed to light irradiation. At certain time intervals, 1 mL of the suspension was extracted and centrifuged (15,000 rpm for 2 min) to remove the photocatalyst. The degradation process was monitored by measuring the UV–visible absorption of Orange II at 485 nm. 

For experiments conducted with immobilized ZnO NRs, 10 glass slides, accounting for a total area of 87.5 cm^2^ and covered by 148.5 mg of photocatalyst, were introduced in a glass Petri dish. Next, 60 mL of the Orange II solution was added and the photodegradation was monitored as previously.

### 2.2. Photocatalytic Inactivation of E. coli

The *E. coli* strain MG1655 [31] was used as model bacteria for all photocatalytic inactivation tests (see the Supplementary Materials for the growth conditions). For each experiment, four conditions were used, namely, with and without photocatalyst, and with and without light irradiation. For the experiments with the dispersed photocalyst, 20 mL of *E. coli* MG1655 cells suspension (10^6^ CFU/mL) was mixed with 20 mg of ZnO NRs (final concentration 1 g/L) in a 100-mL sterile beaker. The bacteria/ZnO NRs mixtures were then either irradiated or kept in the dark for 3 h under stirring. Serial dilutions were performed in sterile demineralized water and aliquots were plated on LB agar for 24 h at 37 °C for the estimation of viable cultivable cells. Each experiment was repeated three times for assessing reproducibility. For the experiments with the immobilized photocatalyst, 260 mg of ZnO NRs was dispersed in 60 mL of demineralized water and immobilized on 10 glass slides. Five of these slides were used for the control experiments conducted in the dark and five for photocatalytic experiments. In both conditions, 50 mL of the bacterial suspension (5 × 10^5^ CFU/mL) was introduced into a Petri dish (ID = 120 mm) containing the ZnO covered glass slides.

### 2.3. Fluorescence Spectroscopy Measurements

Direct interactions between the bacteria proteins and ZnO NRs during photocatalytic experiments were estimated by monitoring the tryptophan (Trp) fluorescence emission at 331 nm following an excitation at 295 nm. Bacteria samples were taken during the photocatalytic experiment and the Trp fluorescence was measured after vortexing.

### 2.4. Estimation of Cell Membrane Integrity by Fluorescence Microscopy

Bacterial cell counts were determined microscopically after SYBR Green II (Thermo Fisher, Courtaboeuf, France) and propidium iodide (PI) staining (Sigma Aldrich, Saint Quentin Fallavier, France). After contact (or not) with dispersed or immobilized NRs, cell suspensions (2 mL) were stained both by SYBR Green II (final concentration 10X) and PI (final concentration 0.6 mM) for 15 min in the dark. After staining, the suspension was filtered through a 25-mm diameter, 0.2-µm pore-size black polycarbonate membrane (Whatman, Piscataway, NJ, USA), air dried, and mounted in mounting oil. Cell counts were carried out with an epifluorescence microscope (BX40, Olympus, Tokyo, Japan) equipped with a ×100 immersion objective lens, a 470–490-nm excitation filter, and a 520-nm barrier filter. Undamaged cells (SYBR-Green-stained cells) and membrane-damaged cells (PI red-stained cells) were enumerated using 60 randomly chosen fields. The results were expressed as cells/mL.

### 2.5. Assessing Cell Alteration by Flow Cytometry

Cells suspensions were washed and resuspended as previously described except that a 0.85% NaCl saline buffer filtered beforehand (0.2 µm) was used to limit contaminating particle impurities. Cells were subsequently stained (or not) with SYBR Green II (10X final concentration) for 15 min and analyzed by flow cytometry (BD Accuri™ C6, BD Biosciences, San Jose, CA, USA) equipped with a 50-mW laser emitting at 488 nm. Commercial distilled apyrogen sterile ultrapure water (B-Braun water, B-Braun, Melsungen, Germany) was used as sheath fluid. Bacteria DNA stained with SYBR Green II fluorescence intensity were collected at FL2 channel (λ_em_: 510 ± 15 nm), as well as forward (FSC) and sideward (SSC) scattered light intensities that give an estimation of the size and granularity of the cells, respectively. Events were triggered on the forward scatter parameter with a threshold of 5000 a.u. and on FL2 with a threshold of 2000 a.u. to narrow the signal to fluorescent cells and to separate positive signals (stained cells) from background. This gating was kept the same for all samples in order to achieve comparable data. The data were analyzed using BD Accuri™ C6 software (BD Biosciences). Event counts provide an estimation of the total cell amount (damaged plus live cells), while the stability of the mean fluorescence reflects the integrity of the intracellular nucleic acid.

### 2.6. Scanning Electron Microscope (SEM) Observations of E. coli Cells

During the photocatalytic inactivation tests of *E. coli*, samples of cell suspensions were centrifuged to remove the NaCl solution and then fixed with 2.5% glutaraldehyde for 2 h. Next, the cells were dehydrated through a graded series of ethanol solutions (37%, 67%, 95%, and 100%) for 15 min and observed using a JSM-6490 LV scanning electron microscope (JEOL, Tokyo, Japan).

### 2.7. E. coli Cell Morphology Analysis by Atomic Force Microscopy (AFM) 

The *E. coli* MG1655 cell morphology was investigated by AFM in intermittent contact mode (Tapping mode) on an Asylum Research MFP-3D Infinity™ equipped with a 100-µm close-loop scanner (Asylum Research, Santa Barbara, CA, USA). Bacterial cell suspensions were deposited on a glass slide and then air dried at room temperature before observation. Point probe robes (Asylum Research, Santa Barbara, CA, USA) were used. The cantilevers had a resonance frequency around 270 kHz and spring constant values around 26 N·m^−1^. Images were acquired with a drive amplitude of 15 mV and an attenuation set-point of 0.8 to 0.7. At least five different zones were scanned on each sample and images at different magnifications were acquired (only 5 × 5 µm^2^ and 1 × 1 µm^2^ images are shown). The images were treated and analyzed using procedures developed under Igor (version Pro 7, Wavemetrics, Asylum, Portland, OR, USA).

## 3. Results

### 3.1. Synthesis and Characterization of ZnO NRs

**Figure 1:**
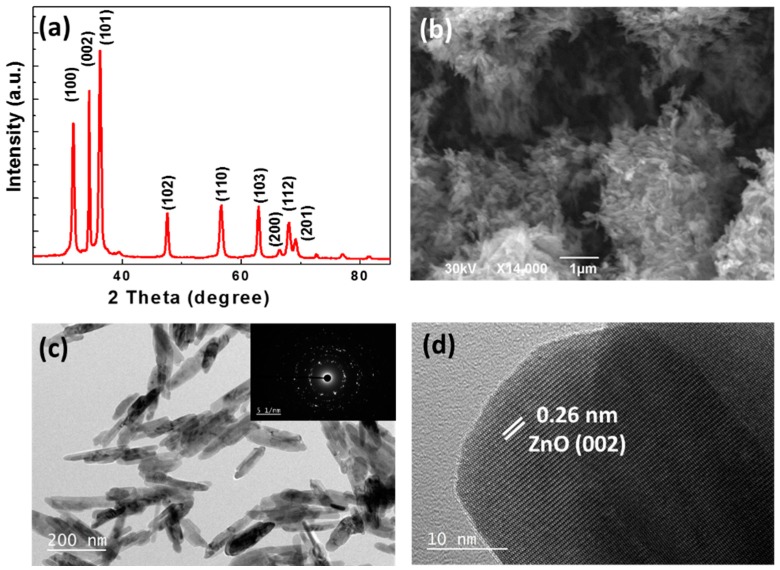
(**a**) X-ray diffraction (XRD) pattern, (**b**) scanning electron microscopy (SEM), and (**c**,**d**) transmission electron microscopy (TEM) images of the ZnO nanorods (NRs) (the inset of Figure 1c is the selected area electron diffraction (SAED) pattern).

ZnO NRs were prepared via a sol–gel process by treatment of Zn(OAc)_2_ with NaOH in ethanol at 60 °C [30]. Figure 1a shows the X-ray diffraction (XRD) pattern of the NRs obtained after 72 h reaction at 60 °C. All diffraction peaks are intense and narrow, which indicates that the NRs are highly crystalline. All peaks correspond to the hexagonal wurtzite structure of ZnO with cell constants of a = 0.3251 nm and c = 0.5208 nm (JCPDS Card No. 36-1451) and no impurities were detected. SEM and transmission electron microscopy (TEM) data collected for the same nanocrystals are shown in Figure 1b,c. Analysis of ca. 100 particles indicates that the ZnO NRs have an average length of 186 nm and a diameter of ca. 20 nm and that their size distribution is relatively narrow. The high crystallinity of the particles is further evidenced from the selected area electron diffraction (SAED) pattern shown in the inset of Figure 1c and from the HR-TEM image that shows continuous lattice fringes through the whole particle (Figure 1d). The interplanar spacing is 0.26 nm and corresponds well to the (002) plane of wurtzite ZnO. The UV–visible diffuse reflectance spectrum of ZnO NRs is shown in Figure S2. The bandgap energy of ZnO NRs (3.25 eV) was determined from their UV–visible diffuse reflectance spectrum and by plotting [F(R)hυ]^2^ vs. photon energy followed by extrapolating the plots at [F(R)hυ]^2^ = 0 (F(R) is the Kubelka–Munk function, h is the Planck constant, and υ is the frequency) (Figure S2). 

**Figure 2:**
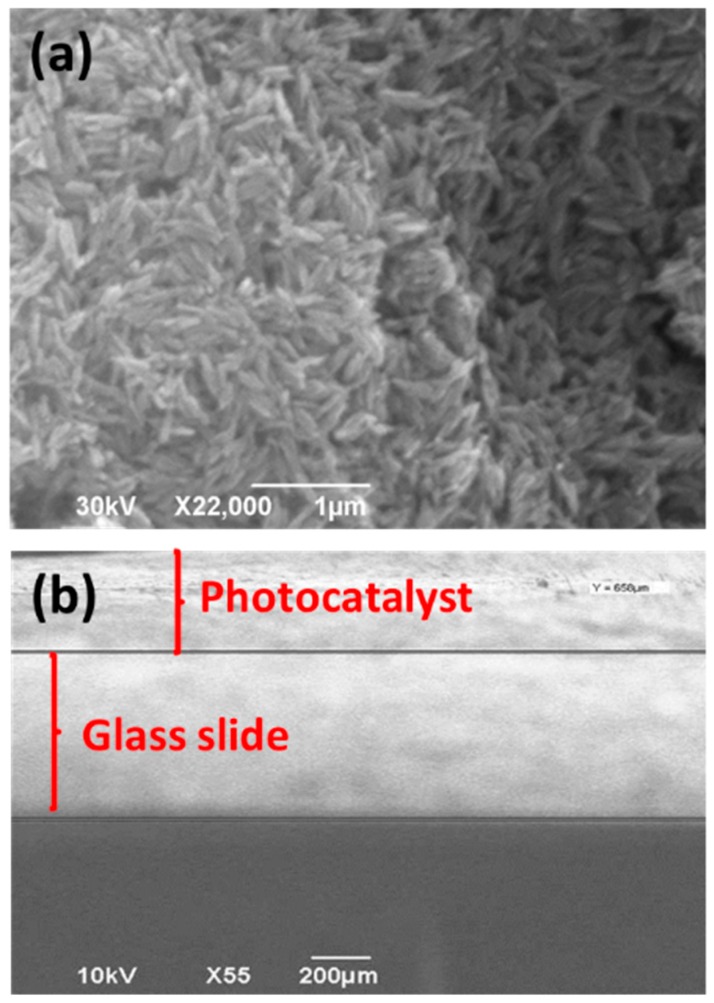
SEM images of the immobilized ZnO photocatalyst (**a**) top view and (**b**) cross sectional view.

For practical applications and to easily reuse the photocatalyst without separation via centrifugation or filtration, the ZnO NRs were immobilized on microscope glass slides. For that purpose, an aqueous dispersion of the ZnO NRs was deposited on glass slides followed by a thermal treatment at 70 °C for 12 h. The SEM images of the immobilized catalyst demonstrate that the size and the morphology of the NRs were not altered by this treatment (Figure 2a). The thickness of the ZnO NR layer deposited was estimated to be 650 µm (Figure 2b).

### 3.2. Photocatalytic Degradation of Orange II

The photocatalytic efficiency of ZnO NRs, used either as powder or as film, was first evaluated in a degradation test of a 10-mg/L Orange II aqueous solution under simulated sunlight irradiation (5.5 mW/cm^2^). Due to their bandgap of 3.25 eV, ZnO NRs can only be photoactivated by light with a wavelength lower than ca. 381 nm. Photocatalytic experiments were conducted under sunlight irradiation to demonstrate that this light source can successfully be used for the degradation of dyes or bacteria (vide infra). For experiments conducted with the dispersed ZnO NRs, 40 mg of photocatalyst was mixed in 40 mL of the Orange II aqueous solution. For the immobilized catalyst, 148.5 mg of the ZnO NRs deposited on glass slides was immersed into 60 mL of the Orange II solution. 

**Figure 3:**
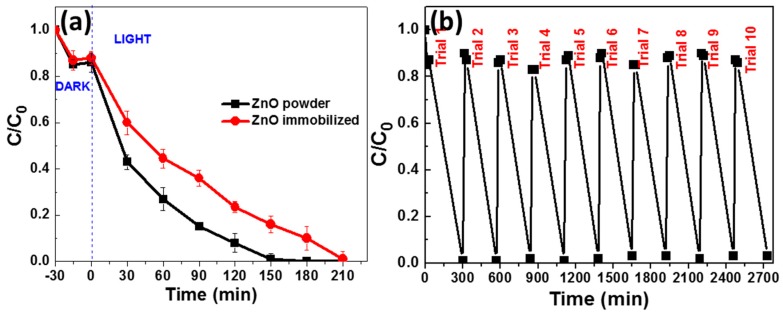
(**a**) Variation of the Orange II concentration as a function of irradiation time (C is the concentration at time t, and C_0_ is the initial concentration). (**b**) Reusability of ZnO NRs fixed on glass slides. Experiments were performed under simulated sunlight irradiation (intensity of 5.5 mW/cm^2^).

Preliminary blank experiments demonstrated that, in the absence of the catalyst, no bleaching of the dye was observed after 5 h of irradiation. Once the photocatalyst was added, and after reaching the adsorption–desorption equilibrium (ca. 30 min, 15% of Orange II adsorbed at the surface of ZnO NRs), a gradual decrease in the dye UV–visible absorption at 484 nm was observed as a function of the irradiation time (Figure S3). The complete disappearance of Orange II was obtained after 150 min irradiation with 40 mg of ZnO NRs used as powder, and in 210 min with the immobilized photocatalyst (Figure 3a). The UV–visible emission spectrum of the lamp used for photocatalytic experiments and the absorption spectrum of Orange II are provided in Figures S1 and S2, respectively. An overlap between the two spectra can be observed, suggesting that the electrons photoexcited from the HOMO to the LUMO of Orange II may be transferred to the CB of ZnO and thus contribute to the photodegradation of the dye.

The efficiencies of Orange II degradation by ZnO NRs were determined quantitatively using the pseudo-first-order model ln (C/C_0_) = −kt, where k is the apparent rate constant (min^−1^) and C_0_ and C are the concentrations of Orange II at time 0 and t, respectively (Figure S4). The rate constants k determined for the bleaching of the dye solution were found to be 0.016 and 0.004 min^−1^ for ZnO NRs used as powder and as film, respectively (results were normalized relative to the mass of ZnO NRs and to the volume of the Orange II solution). The lower photocatalytic activity observed for the immobilized catalyst likely originates from both its lower specific surface compared to dispersed ZnO NRs and the mass transfer limitation, and thus from the reduced probability of contact between the dye and the photocatalyst as reported in studies dedicated to TiO_2_ photocatalysts [32,33,34].

The stability and the reusability of the photocatalyst were investigated using the immobilized ZnO NRs without any washing or drying treatment between two runs (Figure 3b). As can be seen, no significant decrease of catalytic activity could be observed during the 10 successive cycles of Orange II photodegradation. Moreover, XRD and SEM experiments demonstrate that the crystallinity and the morphology of deposited particles were not altered during the repeated experiments (Figures S5 and S6).

### 3.3. Photocatalytic Inactivation of E. coli MG1655 

**Figure 4:**
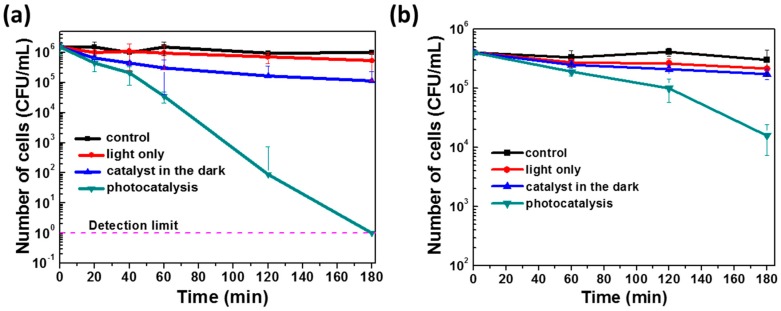
Loss of cultivability of *E. coli* MG1655 (colony forming units) during photocatalysis using (**a**) dispersed and (**b**) immobilized ZnO NRs. “Control” represents a light-free, ZnO-free condition, while “light only” corresponds to light-exposed cells in the absence of the photocatalyst.

Figure 4 shows the bacterial inactivation of suspended *E. coli* MG1655 cells using dispersed and fixed photocatalysts. Control experiments (under light irradiation but in the absence of the photocatalyst or with the photocatalyst used in the dark) are also provided as reference. Simulated sunlight irradiation has no detectable effect on the *E. coli* MG1655 cultivability in the absence of the photocatalyst as seen by growth on nutritive medium. Dispersed and immobilized ZnO NRs used in the dark induce a 10-fold and 3-fold decrease of cell viability after 3 h, respectively, thus indicating that these particles exhibit toxicity towards bacteria even in the absence of light activation. These results are in good agreement with previous reports demonstrating that ZnO particles could be toxic to bacteria even in the dark. This toxicity may originate either from the association of ZnO NRs with the cell surface components leading to structural damages [35,36,37,38] or from the Zn^2+^ ions produced by the partial dissolution of ZnO particles followed by ROS generation [39,40,41]. 

The rates of bacterial inactivation were markedly enhanced when ZnO NRs were used under light irradiation and when increasing the irradiation time (Figure 4). With the dispersed catalyst, the bacterial survival showed a continuous decrease with time and a full cell inactivation (over a 6-log decrease) was observed past 3 h. With immobilized ZnO NRs, the bacterial inactivation was moderate, with a 25-fold survival decrease over a 3-h photocatalysis. As previously observed with Orange II, the photocatalytic activity of the fixed catalyst is lower than that of the dispersed one. The stability of the immobilized photocatalyst during repeated bacterial inactivation was also evaluated. The same catalyst could be reused for five photocatalytic cycles after a simple washing with demineralized water without altering significantly the inactivation rate (from 1.22- to 1.15-log decrease over the five cycles) (Figure S7). 

We finally evaluated the influence of the amount of ZnO NRs immobilized onto the glass slides on the photocatalytic activity (Figure S8). Indeed, the amount of catalyst used is a key parameter in photocatalytic experiments because the pollutants or microorganisms have to be in the close vicinity of the photocatalyst surface in order to be degraded or inactivated. When two glass slides (covered by 52 mg of ZnO NRs) were used instead of five slides (covered by ca. 130 mg of ZnO NRs), the bacterial inactivation was reduced by ca. 10-fold, thus highlighting the key role played by the availability of the catalyst active sites (Figure S8).

### 3.4. Bacterial Protein Interaction with ZnO Nanorods

**Figure 5:**
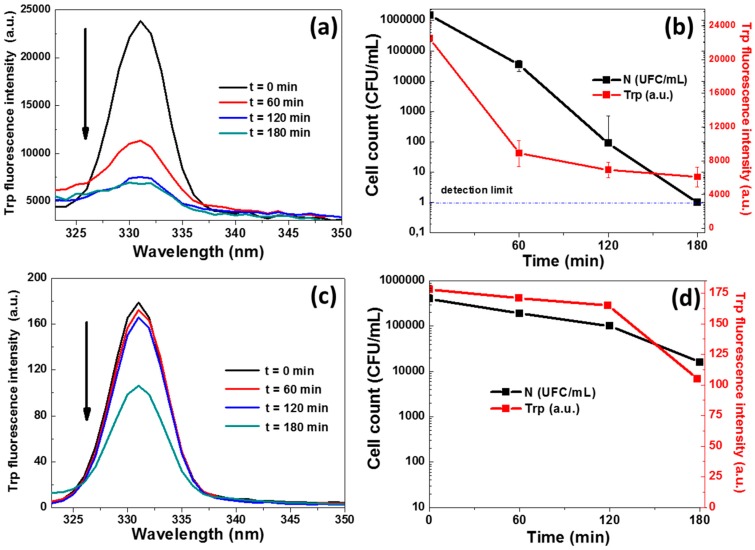
(**a**,**c**) Trp fluorescence emission spectra and (**b**,**d**) time evolution of Trp fluorescence intensity and bacterial cultivability (CFU) during photocatalytic experiments with (**a**,**b**) dispersed and (**c**,**d**) fixed ZnO NRs.

The fluorescence of Trp residues (located at 331 nm after excitation at 295 nm) is very sensitive to its local environment and is often used to report alteration of protein structure/conformation [42,43,44]. In order to better understand the effects of photocatalysis on the loss of bacterial viability, we monitored the evolution of protein fluorescence associated with the Trp residues during photocatalytic experiments. As shown in Figure 5a,c, the intensity of the Trp fluorescence decreases by ca. 3.6-fold and 2-fold during photocatalytic experiments conducted with dispersed and immobilized ZnO NRs, respectively, which indicates changes in the local environment of the Trp residues. Interestingly, the strong decrease of the photoluminescence intensity observed with the dispersed photocatalyst is correlated with the marked losses of bacterial cultivability (colony forming units), thus indicating a close relationship between the local changes of protein conformations and the NR phototoxicity. Control experiments where bacteria were exposed to NRs in the dark showed a much weaker alteration of their Trp fluorescence profiles (Figure S9a in Supplementary Materials File 1). After 1 h of photocatalysis, the Trp fluorescence intensity was decreased by ca. 2.6-fold, while it was only of 1.1-fold when using the catalyst in the dark (Figure S9b in Supplementary Materials File 1). This indicates that local changes in the environment of the bacterial proteins are rather linked to the photocatalytic process and are unlikely to result from a direct bacteria/ZnO NR interaction as demonstrated by the minor decrease of fluorescence observed in the dark (Figure S9). 

### 3.5. Photocatalysis-Induced Cell Surface Alterations 

**Figure 6:**
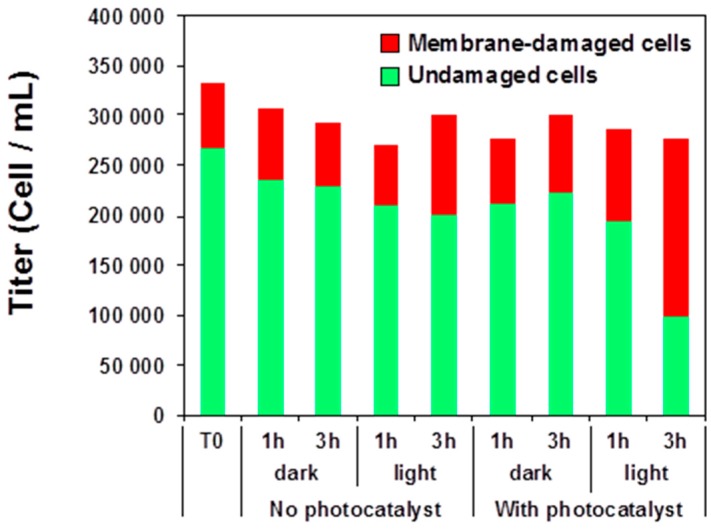
Enumeration of undamaged (SYBR Green II staining) and membrane-altered (propidium iodide (PI) staining) of bacteria by epifluorescence microscopy.

Considering that the decrease of the Trp fluorescence originates from changes in the local environment of some bacterial proteins, which in turn should not occur without cell structure damage, we further explored the effect of photocatalysis on bacterial membrane integrity. A series of experiments using epifluorescence microscopy was performed to analyze the changes induced on the bacterial cell membrane permeability during the photocatalytic treatment. These experiments were based on the dual labeling of bacterial DNA with SYBR Green II and propidium iodide (PI) fluorochromes that differ both in their spectral characteristics and their ability to cross cell membranes. Contrary to SYBR Green II, PI can enter the cell only when the cytoplasmic membrane integrity is compromised. Membrane damages were estimated on cell suspensions in saline buffer, with and without exposure to light, and with and without exposure to ZnO NRs (Figure 6). First, no major effects on membrane integrity could be detected in experiments where bacterial suspensions were exposed (or not) for 1 or 3 h to light irradiation (Figure S10b–e). The usual background of PI-stained cells observed at T0 by epifluorescence microscopy likely originates from an altered physiological state of part of the bacterial inoculum and/or from the preparation-associated damages to the cells by centrifugation, for instance. No significant alteration of bacterial membrane permeability was observed when exposing the bacteria to the immobilized ZnO NRs in the dark (Figure S9f–g). However, after 3 h of photocatalytic treatment, a marked increase of the number of bacteria with altered membrane (PI stained) was detected (64% of membrane-damaged cells vs. 20% in the control), which is in agreement with both the loss of bacterial cultivability and the decrease of Trp fluorescence observed previously (Figure 4 and Figure 5). 

**Figure 7:**
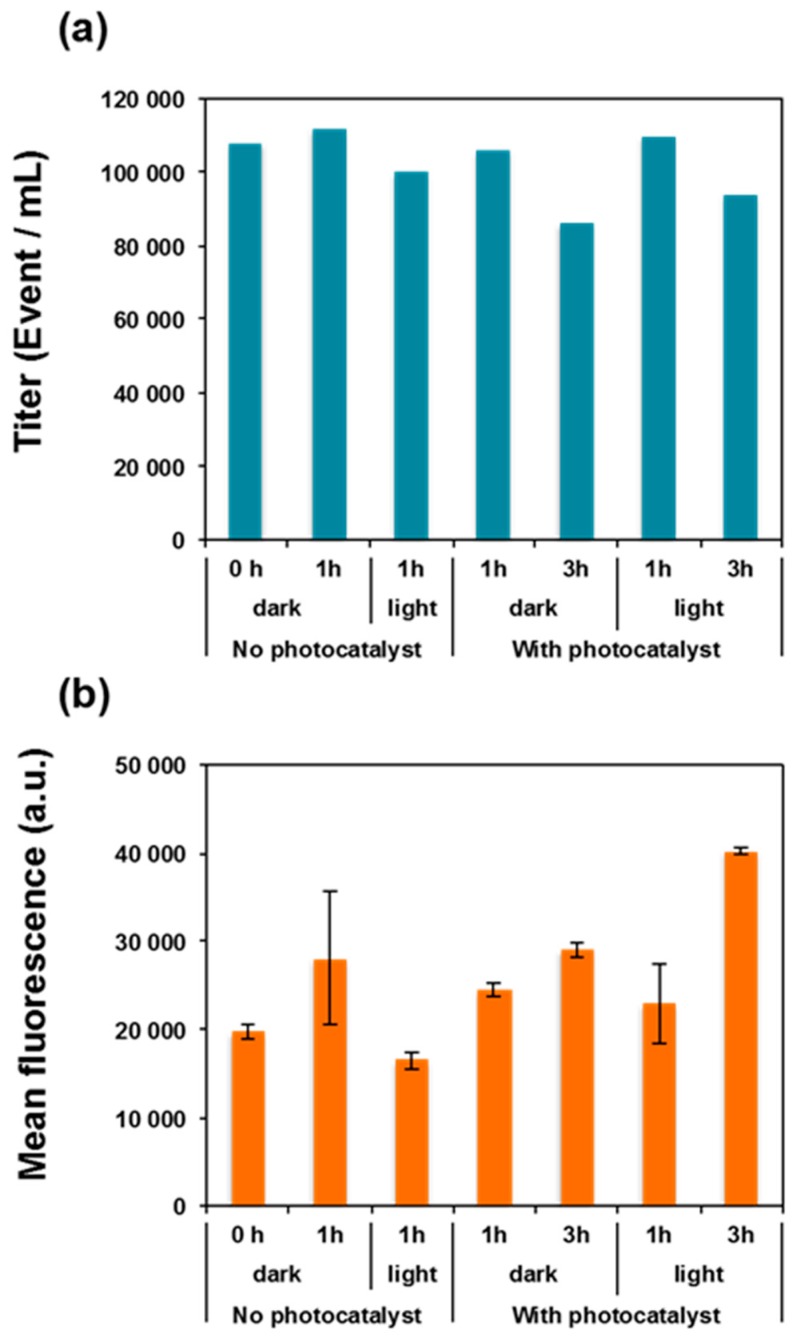
(**a**) Concentration of bacterial cells detected by flow cytometry (FCM) after SYBR Green II staining and (**b**) mean fluorescence intensity of the SYBR Green II fluorochromes per cells. Error bars represent Standard Error of the mean. Each data point represents 1900–3800 events acquired by FCM.

Next, flow cytometry (FCM) was applied in order to quantify SYBR Green II fluorescence in single cells and follow its modifications according to the treatments applied. FCM can be used to discriminate between low- and high-fluorescent bacteria after exposure to stress (chlorine, UV, H_2_O_2_, etc.) [45], revealing an extinction of the fluorescence of the stressed bacteria that may be explained by nucleic acid alteration [46,47]. In particular, this has been demonstrated for pure bacterial strains [48,49,50] and on complex bacterial consortium [51,52]. In this context, *E. coli* cell suspensions were analyzed by FCM after exposure (or not) to ZnO NRs, and with or without light irradiation (cells not exposed to ZnO NRs were used as reference) (Figure 7 and Figure S11). No reduction in the total cell counts was observed but a fraction of the bacteria lost their cultivability, further confirming that the photocatalytic-generated damages are not severe enough to fully disrupt the cells. On the other hand, the analysis of the mean fluorescence intensity of SYBR Green II showed a relatively stable nucleic acid staining, indicating that no detectable alteration of nucleic acids took place during the photocatalytic experiment as implemented. However, the intracellular levels of fluorescence were statistically compared two by two using a standard Z-test. None of the conditions produced a significant difference when the 1-h exposure time was analyzed. Small but significant differences were observed when comparing two incubation times for a given condition (1 vs. 3 h; *p* < 10^−4^) or for the effect of light irradiation (dark versus light) but only at 3 h of irradiation (*p* < 10^−4^). Altogether, these results suggest that the photocatalytic treatment mostly injured surface structures on *E. coli* cells, which resulted in altered cells that progressively lost their viability. 

### 3.6. Photocatalysis-Induced Cell Structure Damages

**Figure 8:**
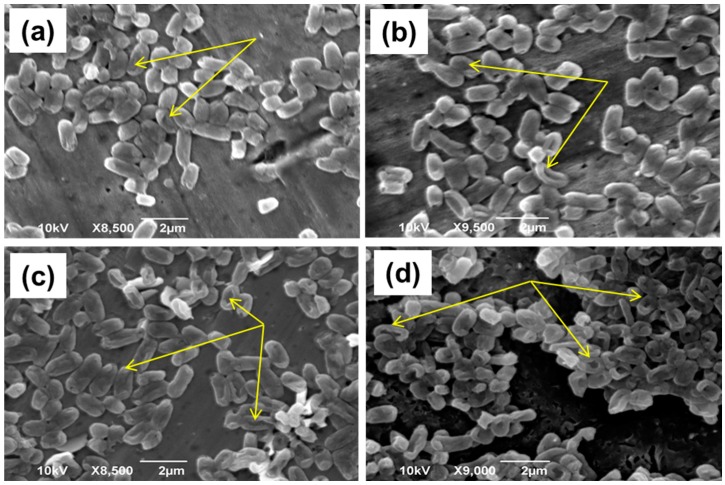
SEM images of *E. coli* MG1655 bacteria (**a**,**b**) exposed to the photocatalyst in the dark for 1.5 and 3 h, respectively, and (**c**,**d**) exposed to the photocatalyst under light irradiation for 1.5 and 3 h, respectively. The arrows indicate some of the most visible damaged cells.

Morphological alterations of *E. coli* cells (10^8^ CFU/mL), generated by exposure to ZnO NRs under light irradiation or in the dark, were examined by SEM. Control *E. coli* cells appeared as rod-shaped cells with an average size varying between 1 and 2 µm, with surfaces that were continuous and did not present visible alterations (data not shown). After 1.5 or 3 h of contact with the ZnO NRs in the dark, some *E. coli* cells displayed morphological changes that mostly appeared as flattened cells (Figure 8a,b). Meanwhile, after the photocatalytic treatment (Figure 8c,d), the majority of cells display morphological alterations with a concomitant cell size reduction that increases in a time-dependent manner. These results are in accordance with previous reports indicating the cell envelope as a primary target of the ROS generated during photocatalysis [53,54]. 

**Figure 9:**
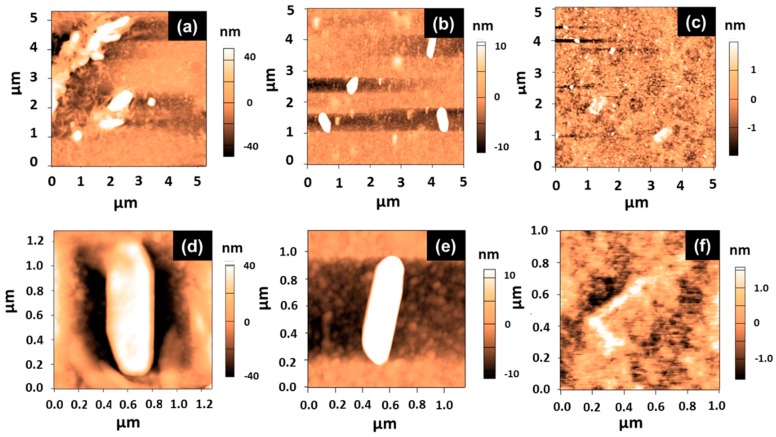
Atomic force microscopy (AFM) images of *E. coli* MG1655. (**a**–**c**) Height images at large scan and (**d**–**f**) height images at small scan (**a**,**d**) before, (**b**,**e**) after 1.5 h, and (**c**,**f**) after 3 h of photocatalysis.

AFM analyses were also carried out to further support the ZnO-NR-generated morphological alteration on *E. coli* under light irradiation. It is noteworthy that these experiments were conducted using a cell concentration of 5 × 10^5^ CFU/mL (10^8^ CFU/mL was used in SEM experiments) and thus the damages caused to cells should be more visible than those observed by SEM analyses. Figure 9 shows AFM topography images of *E. coli* cells before photocatalysis (Figure 9a,d) at different magnifications, from which no surface defect could be observed (Figure 9d). The morphometric measurement realized on the typical bacterial cells displayed in Figure 9d showed an expected size of 1 μm, with a ca. 400-nm width and a ca. 85-nm height. After 1.5 h of photocatalysis using fixed ZnO NRs, *E. coli* cells remain detectable but their rod-shape started to disappear (Figure 9b), and the size of the bacteria decreased, as shown on a randomly selected cell in Figure 9e (i.e., 800-nm length, 200-nm width, and 40-nm height). Finally, after 3 h of photocatalysis (Figure 9c,f), a complete lysis of the cells was observed and only thin bacterial fragments could be detected. Measurements of one of these fragments show an object with a 200-nm length, a 2-nm width, and a 2-nm thickness, which suggests an empty lysed cell. 

Our investigations demonstrate that photocatalysis mediated by ZnO NRs mainly results in a succession of surface alterations (membrane damages, altered morphology) and that DNA is not affected. It should be noted that the alterations observed by fluorescence microscopy and FCM were not as severe as those observed by SEM and AFM. We attribute these differences to the cell sample preparation. Indeed, SEM and AFM required the samples to be dried out for observation, which is a relatively harsh condition for bacterial cells that went through a photocatalysis that damaged their surfaces. Considering that the bacterial cell envelope is responsible for its shape and it resistance to osmotic pressure, it is very likely that ZnO-injured bacterial surfaces could not maintain the cell structure under drying conditions, which appears as cell lysis in AFM or an altered morphology of glutaraldehyde-fixed cells in SEM. Nevertheless, the most dramatic effect of photocatalysis observed by SEM and AFM results from the same surface alteration of the bacterial cells.

## 4. Conclusions

ZnO NRs with an average length and diameter of 186 and 20 nm, respectively, were easily prepared via a low-temperature sol–gel process. These nanocrystals were found to exhibit high solar photocatalytic activity both in dispersed form or immobilized on glass for the photodegradation of the Orange II organic dye and for the *E. coli* bacteria decontamination. Although less photocatalytically active than the dispersed ZnO NRs, the immobilized rods can be easily reused at least five times for bacterial inactivation without a significant decrease in activity. Fluorescence spectroscopy, epifluorescence microscopy, SEM, and AFM provide evidence that photocatalytic treatment alters *E. coli* bacterial surfaces, resulting in the inactivation of the bacteria, and that inactivation occurs before intracellular damage occur, as no damage of nucleic acid could be detected. Our results demonstrate that photocatalysis using ZnO NRs constitutes an alternative to classical oxidation treatments such as chlorination and ozonization, especially in isolated areas, and that ZnO NRs have the potential to be used within real environmental applications.

## Supplementary Materials

The following are available online at http://www.mdpi.com/1996-1944/11/11/2158/s1, Figure S1: UV–visible absorption spectrum of the light source used for photocatalytic experiments, Figure S2: (a) Room-temperature UV–visible diffuse reflectance spectra of ZnO NRs. (b) Tauc plot of ZnO NRs for the determination of the bandgap energy value, Figure S3: UV–visible spectra used to monitor the variation of the Orange II concentration as a function of irradiation time using ZnO NRs as (a) powder and (b) immobilized on glass slides, Figure S4: Plots of ln(C_0_/C) vs. reaction time for the ZnO photocatalyst used as powder or deposited on glass slides, Figure S5: XRD patterns of the immobilized ZnO NRs (a) before and (b) after 10 successive photocatalytic cycles, Figure S6: SEM images of the immobilized ZnO NRs (a) before and (b) after 10 successive photocatalytic cycles, Figure S7: Reusability of immobilized ZnO NRs in photocatalytic bacterial inactivation, Figure S8: Influence of the amount of immobilized ZnO NRs used on the inactivation of *E. coli* MG1655; (a) 130 mg and (b) 52 mg of the photocatalyst were used, respectively, Figure S9: Time evolution of (a) Trp fluorescence emission spectra when mixing *E. coli* cells with ZnO NRs in the dark and (b) Trp fluorescence intensity when mixing *E. coli* cells with fixed ZnO NRs in the dark or under light illumination, Figure S10: Photography of the *E. coli* preparation under epifluorescence microscope after bacterial staining with SYBR Green II (impermeant membranes—green fluorescence) and propidium iodide (permeabilized membrane—red fluorescence). (a) Initial bacterial suspension, (b,c) control after 1 and 3 h, respectively, (d,e) bacteria exposed to light after 1 and 3 h, respectively, (f,g) bacteria exposed to ZnO NRs in the dark after 1 and 3 h, respectively, and (h,i) bacteria exposed to fixed ZnO NRs and to light irradiation after 1 and 3 h, respectively, Figure S11: Representative cytograms of *E. coli* populations exposed or not to ZnO NRs before and after staining the bacterial cells with SYBR Green II fluorochromes. P1, P2, and P3 represent the gating zones illustrative of: bacterial signal before and after staining and signal parasite of control NaCl + ZnO NRs without bacteria, respectively.
