# Supplementary material for: ZnO Nanorods with High Photocatalytic and Antibacterial Activity under Solar Light Irradiation
Source: Materials (Basel). 2018 Nov 1;11(11):2158. doi: 10.3390/ma11112158 (PMC6266891; doi:10.3390/ma11112158)
Supplement: Supplementary file 1 [file materials-11-02158-s001.pdf]

Supplementary Materials: ZnO Nanorods with High Photocatalytic and Antibacterial Activity under Solar Light Irradiation

Faouzi Achouri, Christophe Merlin, Serge Corbel, Halima Alem, Laurence Mathieu, Lavinia Balan, Ghouti Medjahdi, Myriam Ben Said, Ahmed Ghrabi, and Raphaël Schneider

Synthesis of ZnO NRs

Materials

$Zn(OAc)_2$. $2H_2O$ (> 98%, Sigma), Orange II sodium salt (> 85%, Sigma), sodium hydroxide (> 97%, Sigma), anhydrous methanol (Sigma), and absolute ethanol (HPLC Grade) were used as received without further purification. All solutions were prepared using Milli-Q water (18.2 MΩ.cm, Millipore) as a solvent.

Synthesis of ZnO NRs and Immobilization onto Glass Slides

A sol–gel solvothermal process using $Zn(OAc)_2$ as the starting material was used to prepare ZnO NRs [1]. In a typical synthesis, $Zn(OAc)_2$. $2H_2O$ (7.37 g, 3.35 mmol) and NaOH (3.7 g, 9.25 mmol) were separately dissolved in 16 mL of methanol. Next, the NaOH solution was added dropwise to the $Zn(OAc)_2$. $2H_2O$ solution and the mixture was stirred for 72 h at 60 °C. After cooling to room temperature, the ZnO NRs were collected by centrifugation, washed three times with water, twice with ethanol, and dried at 50 °C overnight. To fix ZnO NRs onto microscope glass slides, 200 mg of the photocatalyst was dispersed in 50 mL of demineralized water and drops of the dispersion were spotted onto glass slides followed by drying in an oven at 70 °C. This operation was repeated until an appropriate mass of catalyst and homogeneous layers were obtained. For photocatalytic experiments conducted with the Orange II dye, 148.5 mg of ZnO NRs were deposited onto 10 glass slides accounting for a total area of 87.5 cm². For experiments conducted with *E. coli*, 260 mg ZnO NRs was deposited onto 10 microscope glass slides. Five of these slides were used for photocatalytic experiments and the other five for control experiments conducted in the dark.

Characterization of ZnO NRs

Transmission electron microscopy (TEM) images were taken by placing a drop of the particles dispersed in methanol onto a carbon-film-supported copper grid. Samples were studied using a Philips CM200 instrument operating at 200 kV. Scanning electron microscopy (SEM) pictures were prepared using a JEOL scanning electron microscope JSM-6490 LV. The X-ray powder diffraction (XRD) diagrams of all samples were measured using Panalytical X'Pert Pro MPD diffractometer using Cu-Kα radiation. The X-ray powder diffraction data were collected from an X'Pert MPD diffractometer (Panalytical AXS) with a goniometer radius of 240 mm, fixed divergence slit module (1/2° divergence slit, 0.04 rd Sollers slits), and an X'Celerator as a detector. The powder samples were placed on a silicon zero-background sample holder and the XRD patterns were recorded at room temperature using Cu-Kα radiation (λ = 0.15418 nm). All the optical measurements were performed at room temperature (20 ± 1 °C) under ambient conditions. Absorption spectra of liquid samples were recorded on a Thermo Scientific Evolution 220 UV–visible spectrophotometer. Photoluminescence (PL) spectra were recorded on a Horiba Fluoromax-4 Job in Y von spectrofluorimeter. The diffuse reflectance absorption spectra (DRS) were recorded on a Shimadzu 2600 UV–visible spectrophotometer. $BaSO_4$ powder was used as a standard for baseline measurements and spectra were recorded in a range of 250–1400 nm.

Preparation of Bacterial Strains and Growth Media

The *E. coli* strain MG1655 [2] was used as model bacteria for all photocatalytic inactivation tests. Bacteria were routinely cultured in Lysogenic Broth (LB) (LB Broth Miller, Difco™) at 37 °C under aerobic conditions with constant stirring at 160 rpm. Bacterial growth was monitored by measuring the optical density of the cultures at 600 nm (OD_{600}). For ZnO NR toxicity testing, bacteria were cultured overnight (≈ 16 h) up to stationary phase, collected by centrifugation at 9000× *g* for 4 min, and then washed twice with sterile demineralized water. Cell pellets were finally resuspended in sterile saline buffer (NaCl 0.85%) and the cell density was adjusted spectrometrically (OD_{600}) to a final cell concentration of 5.10^5–10^6 colony forming units per mL (CFU/mL) for photocatalytic experiments.

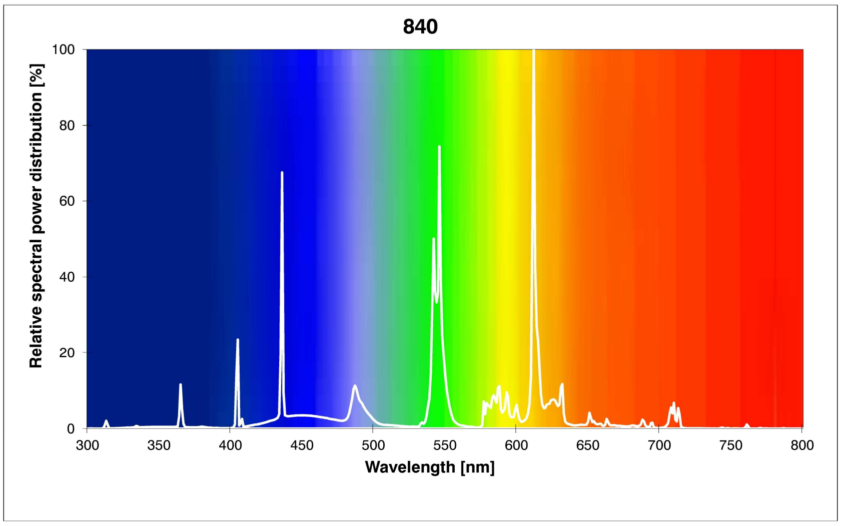

Figure S1. UV–visible absorption spectrum of the light source used for photocatalytic experiments.

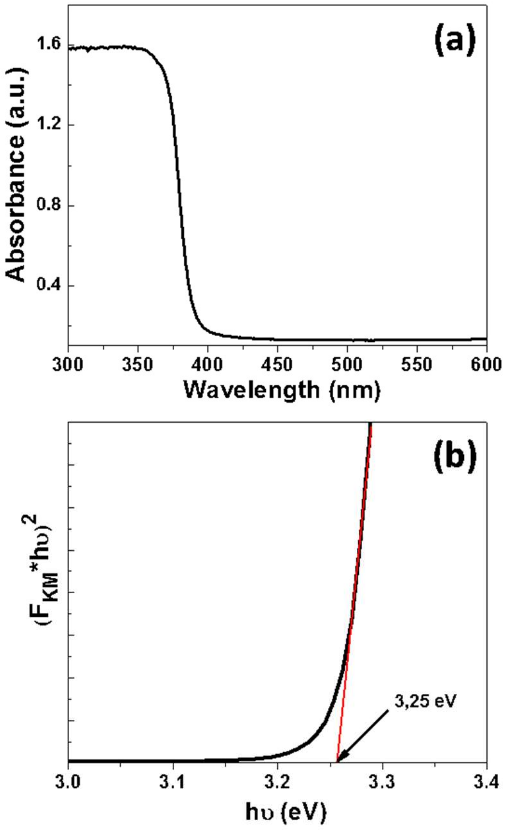

Figure S2. (**a**) Room-temperature UV–visible diffuse reflectance spectra of ZnO NRs. (**b**) Tauc plot of ZnO NRs for the determination of the bandgap energy value.

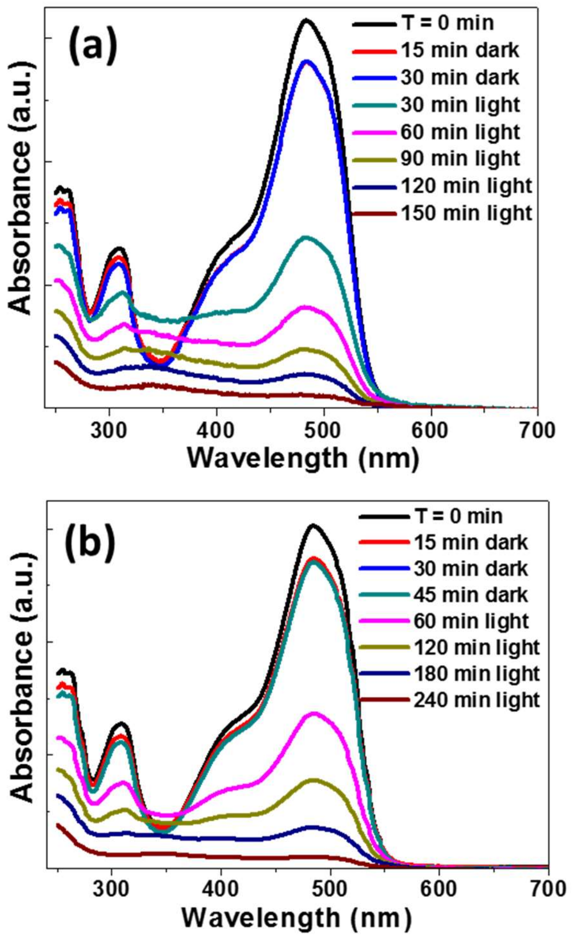

Figure S3. UV–visible spectra used to monitor the variation of the Orange II concentration as a function of irradiation time using ZnO NRs as (**a**) powder and (**b**) immobilized on glass slides.

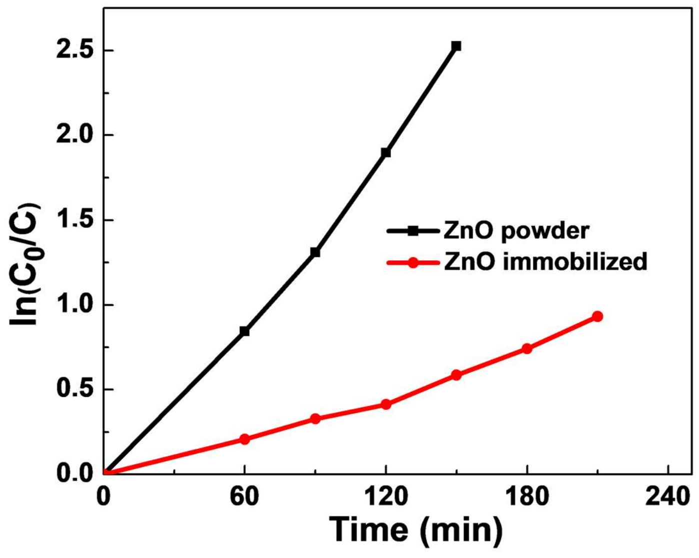

Figure S4. Plots of ln(C_0/C) vs. reaction time for the ZnO photocatalyst used as powder or deposited on glass slides.

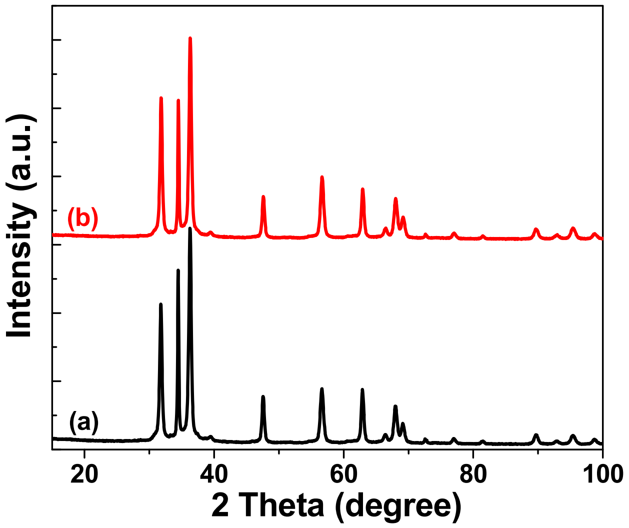

Figure S5. XRD patterns of the immobilized ZnO NRs (**a**) before and (**b**) after 10 successive photocatalytic cycles.

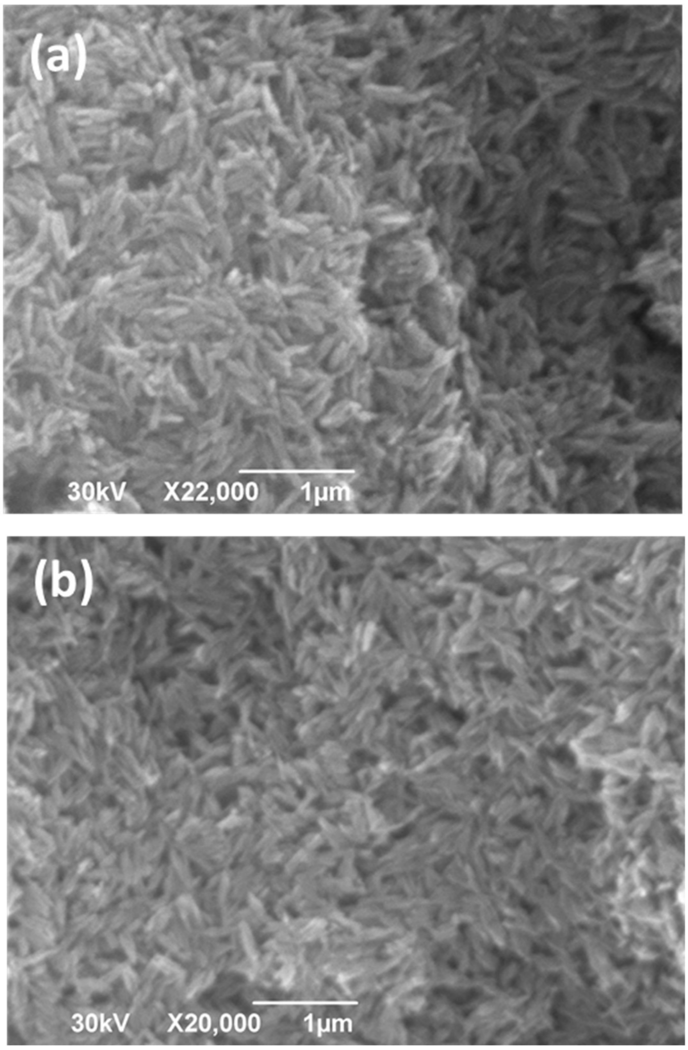

Figure S6. SEM images of the immobilized ZnO NRs (**a**) before and (**b**) after 10 successive photocatalytic cycles.

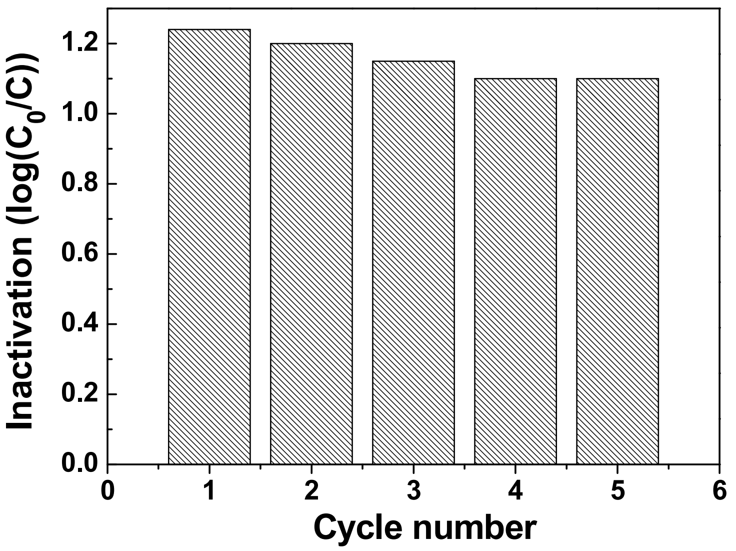

Figure S7. Reusability of immobilized ZnO NRs in photocatalytic bacterial inactivation.

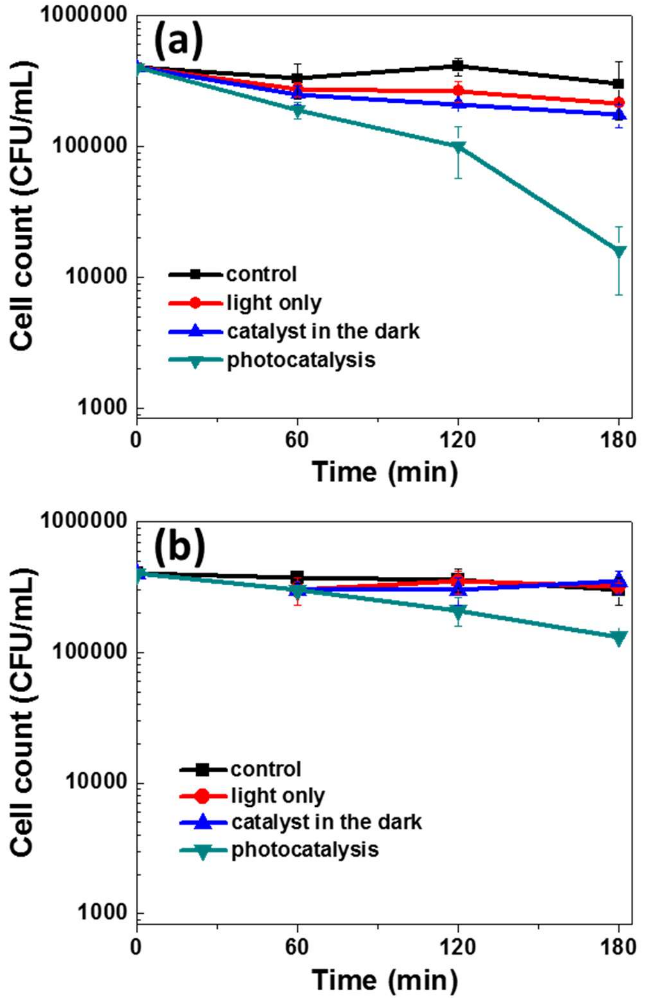

Figure S8. Influence of the amount of immobilized ZnO NRs used on the inactivation of *E. coli* MG1655; (**a**) 130 mg and (**b**) 52 mg of the photocatalyst were used, respectively.

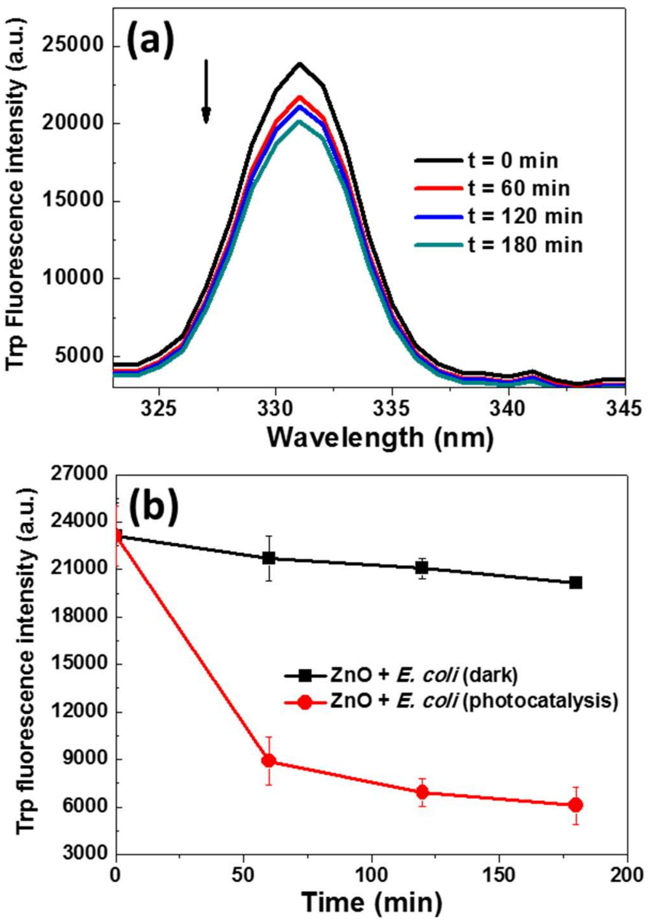

Figure S9. Time evolution of (**a**) Trp fluorescence emission spectra when mixing *E. coli* cells with ZnO NRs in the dark and (**b**) Trp fluorescence intensity when mixing *E. coli* cells with fixed ZnO NRs in the dark or under light illumination.

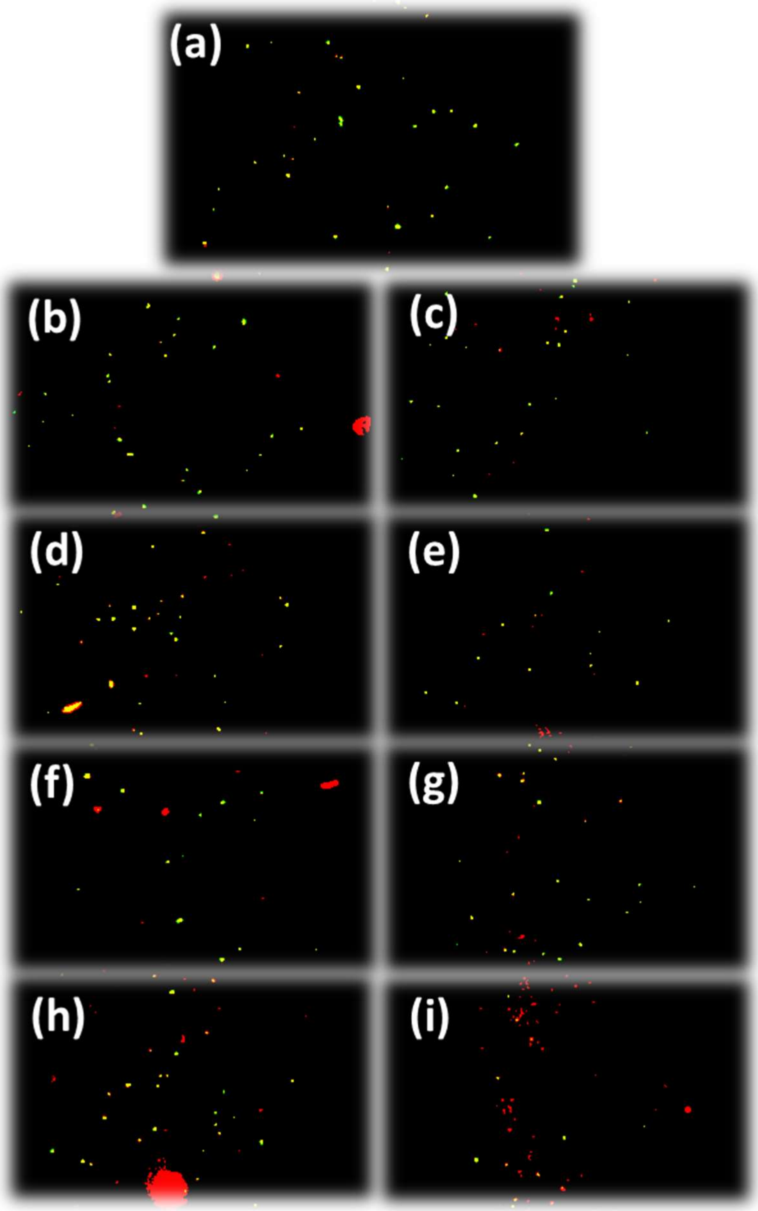

Figure S10. Photography of the *E. coli* preparation under epifluorescence microscope after bacterial staining with SYBR Green II (impermeant membranes—green fluorescence) and propidium iodide (permeabilized membrane—red fluorescence). (**a**) Initial bacterial suspension, (**b**,**c**) control after 1 and 3 h, respectively, (**d**,**e**) bacteria exposed to light after 1 and 3 h, respectively, (**f**,**g**) bacteria exposed to ZnO NRs in the dark after 1 and 3 h, respectively, and (**h**,**i**) bacteria exposed to fixed ZnO NRs and to light irradiation after 1 and 3 h, respectively.

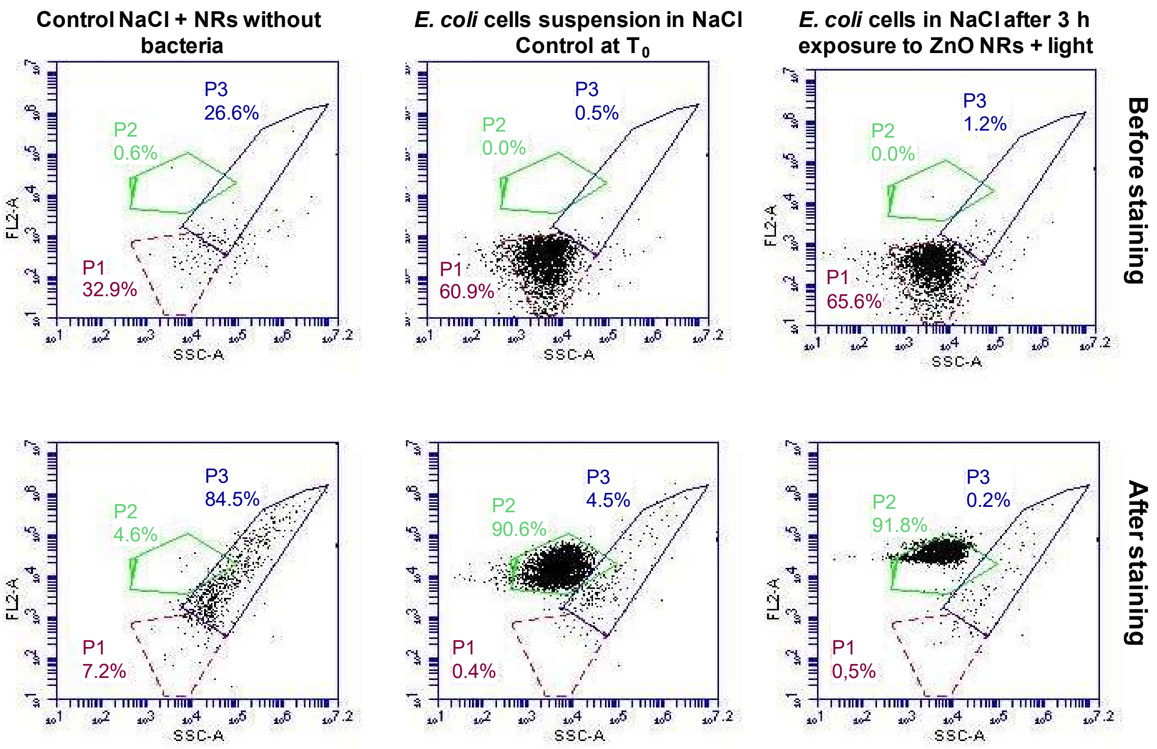

Figure S11. Representative cytograms of *E. coli* populations exposed or not to ZnO NRs before and after staining the bacterial cells with SYBR Green II fluorochromes. P1, P2, and P3 represent the gating zones illustrative of: bacterial signal before and after staining and signal parasite of control NaCl + ZnO NRs without bacteria, respectively.

References

1. Guo, Y.; Wang, H.; He, C.; Qiu, L.; Cao, X. Uniform Carbon-Coated ZnO Nanorods: Microwave-Assisted Preparation, Cytotoxicity, and Photocatalytic Activity. *Langmuir* **2009**, *25*, 4678–4684.
2. Blattner, F.R.; Plunkett, G.; Bloch, C.A.; Perna, N.T.; Burland, V.; Riley, M.; Collado-Vides, J.; Glasner, J.D.; Rode, C.K.; Mayhew, G.F.; et al. The complete genome sequence of *Escherichia coli* K-12. *Science* **1997**, *277*, 1453–1462
